# A Case Report on Biot's Breathing: An Early Sign of Brainstem Dysfunction in Neonatal Bacterial Meningitis

**DOI:** 10.7759/cureus.113465

**Published:** 2026-07-27

**Authors:** Shadi Badr El Din, Chantal Lecart, Antoine Bachy

**Affiliations:** 1 Pediatrics, UCLouvain, Brussels, BEL; 2 Neonatology, Grand Hôpital de Charleroi, Site Les Viviers, Charleroi, BEL

**Keywords:** ataxic respiration, biot's breathing, brainstem dysfunction, neonatal bacterial meningitis, neonatal seizures, post-infectious hydrocephalus, siadh

## Abstract

Neonatal seizures are a diagnostic challenge that requires urgent identification of the underlying etiology. We present the case of a 16-day-old newborn admitted with afebrile seizures and severe hyponatremia, in whom further investigation revealed *Escherichia coli *bacterial meningitis complicated by syndrome of inappropriate antidiuretic hormone secretion (SIADH), post-infectious hydrocephalus, and Biot's breathing. This case highlights the complexity of neonatal seizure evaluation when initial clinical findings are misleading and emphasizes the value of recognizing Biot's breathing as an early clinical sign of brainstem dysfunction in the context of neonatal bacterial meningitis, warranting prompt, multidisciplinary management.

## Introduction

Neonatal seizures are the most common neurological emergency of the newborn period and remain a significant diagnostic and therapeutic challenge, given the immaturity of the neonatal brain and the frequent dissociation between clinical and electrographic events, whereby physical movements may not correlate with underlying electrical seizure activity [1]. Their underlying etiology is diverse and can be difficult to establish when the initial clinical presentation is misleading. Central nervous system infection is an important, potentially treatable cause of neonatal seizures. *Escherichia coli* remains a leading pathogen in neonatal bacterial meningitis, particularly in late-onset disease, and is associated with substantial morbidity, including seizures, hydrocephalus, and long-term neurodevelopmental sequelae [2]. Hyponatremia secondary to syndrome of inappropriate antidiuretic hormone secretion (SIADH) is a well-recognized complication of bacterial meningitis in children, correlating with the severity of meningeal inflammation [3]; severe hyponatremia can itself precipitate metabolic seizures, further complicating the clinical assessment of neonatal seizures of infectious origin. While these infectious and metabolic derangements are increasingly well-characterized, they do not capture the full spectrum of neurological compromise that may accompany severe meningitis, and subtler markers of brainstem involvement are correspondingly easy to overlook.

Beyond these more commonly recognized complications, subtle changes in respiratory pattern can provide an early clue to evolving brainstem dysfunction. Biot’s breathing, first described by Camille Biot in 1876 in a patient with tuberculous meningitis under the term ‘rythme méningitique’ [4], is characterized by irregular respiratory cycles of variable depth interrupted by unpredictable apneic pauses, distinct from the crescendo-decrescendo pattern of Cheyne-Stokes respiration [5]. Despite this historical link to meningeal disease, the sign is rarely referenced in contemporary literature and is exceptionally seldom reported in the neonatal population [5]. To our knowledge, its occurrence in the specific context of neonatal bacterial meningitis has not been described in detail, a gap that the present case seeks to address. Because the neural circuits generating respiratory rhythm reside within the brainstem, alterations in breathing pattern can serve as an early external signal of brainstem compromise, often preceding more overt neurological deterioration. Despite its historical association with meningitis, Biot's breathing is rarely referenced in the contemporary literature, in part because of terminological overlap with “ataxic” or “cluster” breathing, and because widespread early mechanical ventilation now limits clinicians' exposure to spontaneous pathological breathing patterns [5]. In the neonatal population specifically, reports describing this finding are exceptional.

We report the case of a 16-day-old newborn with afebrile seizures, severe hyponatremia, and bacterial meningitis complicated by post-infectious hydrocephalus, in whom the emergence of Biot's breathing served as a clinical marker of brainstem involvement and prompted urgent multidisciplinary escalation of care. This case illustrates the diagnostic complexity of neonatal seizures when initial findings are misleading and underscores the importance of recognizing this rare but clinically significant respiratory pattern.

## Case presentation

A female infant was born at 37 weeks and 4 days of gestation, after a pregnancy marked by suspected intrauterine growth restriction, with a birth weight of 2400 g (10th percentile) and an uneventful early neonatal course. At 16 days of life, she was presented to the emergency department with a 24-hour history of multiple afebrile seizures occurring several times per hour, characterized by loss of consciousness, asymmetric clonic movements, and pallor, each lasting 15-30 seconds. Poor weight gain had been noted in the preceding days, without overt feeding difficulties.

On admission, the infant was pale and hypotonic, with hypersalivation, mild cyanosis, and a newly noted grade 1-2/6 systolic murmur; she was eupneic in ambient air. Vital signs showed tachycardia (heart rate 171/min), oxygen saturation 96% in room air, and temperature 37°C. During peripheral venous access placement, a further seizure occurred with loss of contact, peripheral cyanosis, and generalized hypertonia lasting under one minute.

Initial blood gas analysis revealed severe hyponatremia (117 mmol/L) and preserved acid-base status (pH 7.41). A borderline clitoral hypertrophy initially raised suspicion for congenital adrenal hyperplasia; this finding was not confirmed on repeat examination or biological workup. The infant was admitted to the neonatal intensive care unit for stabilization, blood and cerebrospinal fluid (CSF) sampling, and initiation of empirical antiseizure and antimicrobial therapy (amoxicillin, cefotaxime, amikacin, and aciclovir), together with an elective, preventive intubation to secure the airway in the context of ongoing status epilepticus and the anticipated need for sequential anticonvulsant loading doses (phenobarbital, phenytoin, midazolam), which carry a risk of respiratory depression; she was extubated on day 3 once seizure control was achieved.

**Figure 1:**
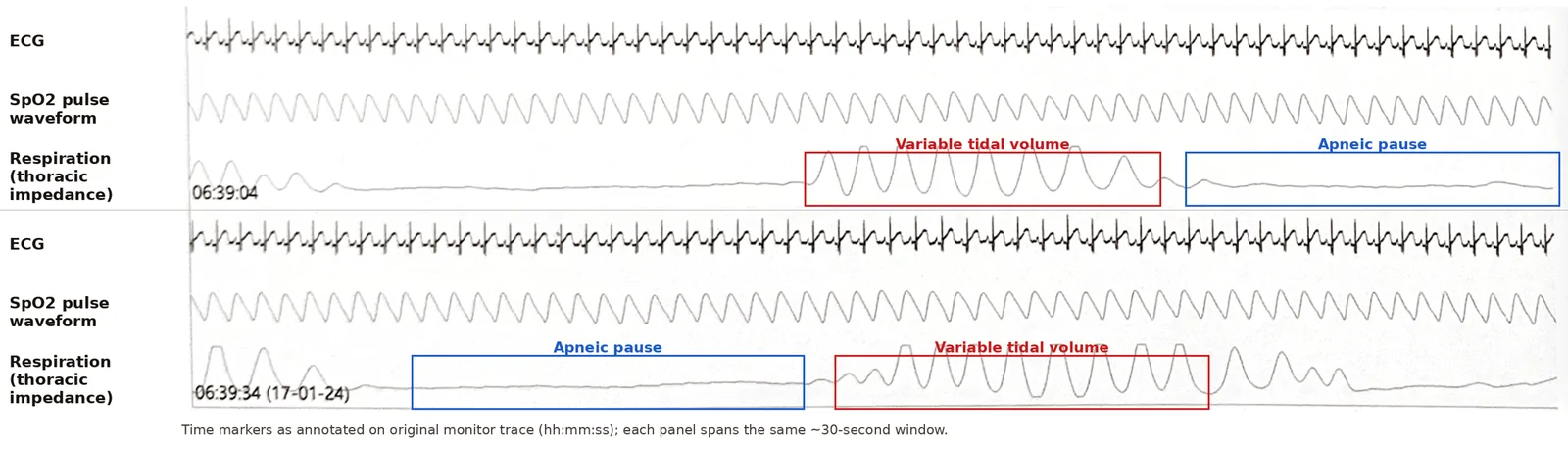
Respiratory monitoring showing an irregular respiratory pattern consistent with Biot's breathing Continuous bedside monitoring in the neonatal intensive care unit, showing (top to bottom in each panel) the ECG, SpO2 pulse waveform, and thoracic impedance respiration trace, recorded at 2 time points 30 seconds apart (06:39:04 and 06:39:34 on 17/01/2024). The respiration trace shows a cluster of breaths of variable tidal volume (red boxes) followed by an apneic pause (blue boxes), without the crescendo–decrescendo modulation of Cheyne–Stokes respiration, consistent with Biot's breathing; the ECG and SpO2 pulse waveform remain regular throughout, indicating that the irregularity is specific to the respiratory trace rather than a monitoring artifact.

Laboratory workup disclosed a marked inflammatory syndrome (initial C-reactive protein (CRP) 133 mg/L) and CSF findings consistent with bacterial meningitis: 2480 nucleated cells/mm³ (70% neutrophils, 8% lymphocytes, 20% histiocytes), 20 red blood cells/mm³, glucose <5 mg/dL against a concurrent serum glucose of 165mg/dL (CSF: serum glucose ratio <0.03), and gram-negative bacilli on direct examination; the sample volume was insufficient for CSF protein, lactate, chloride, or multiplex PCR testing. Standard aerobic blood and CSF cultures, both positive within 24 hours, confirmed *Escherichia coli *bacteremia and meningitis; antiviral therapy was discontinued after 24 hours once herpes simplex virus infection was excluded. Susceptibility testing (resistant to ampicillin and amoxicillin-clavulanate; susceptible to amikacin and cefotaxime) allowed narrowing to cefotaxime monotherapy on day 2. Despite initial biological improvement, the inflammatory syndrome rebounded sharply by day 5 (CRP 424 mg/L), prompting reinstitution of triple antibiotic therapy (cefotaxime, ciprofloxacin, and amikacin). During this period of clinical deterioration, continuous cardiorespiratory monitoring captured an increasingly irregular respiratory pattern, with variable tidal volumes and intermittent apneic pauses lacking any crescendo-decrescendo amplitude modulation, consistent with Biot's breathing (Figure 1).

Cerebral magnetic resonance imaging performed on day 3 demonstrated multiple pericerebellar collections consistent with empyema and marked hydrocephalus with obstruction at the level of the aqueduct of Sylvius. Given the progressive clinical picture - worsening hypertonia and discomfort together with the new respiratory pattern - the first external ventricular drain (EVD) was placed on day 5 by the neurosurgical team, without intrathecal antibiotics; subsequent CSF cultures remained sterile, and the drain was removed on day 12. A marked worsening of hydrocephalus on serial cranial ultrasound and repeat imaging, indicating recurrent aqueductal obstruction despite the first drain, over the following 48 hours required placement of a second EVD on day 14. Given the impracticality of prolonged external drainage, a Rickham reservoir was placed on day 25. Once CSF protein content had normalized sufficiently, a definitive ventriculoperitoneal shunt with a gravitational valve was placed on day 38.

Concurrently, the severe hyponatremia, attributed to SIADH secondary to the central nervous system infection, was corrected over the first 12 hours with intravenous sodium supplementation, which was progressively weaned and discontinued by day 11. Antibiotic therapy was continued as a cefotaxime-ciprofloxacin combination for 21 days from the first sterile CSF sample, then as cefotaxime monotherapy, for a total planned duration of six weeks; CRP normalized by hospital day 20.

Acute seizure control was achieved after approximately 6 hours of treatment with sequential loading doses of phenobarbital, phenytoin, and midazolam, corresponding to an estimated 24-hour period of status epilepticus; because a formal electroencephalogram (EEG) was not technically feasible during the acute event, the response to treatment was guided clinically and by continuous cerebral function monitoring. A first standard EEG, performed on day 3, showed a continuous irritative theta background followed by a discontinuous, burst-suppression pattern with a hypovoltaged, poorly differentiated background, without evidence of ongoing seizures. Maintenance therapy was transitioned to levetiracetam from day 4 onward. No further clinical or electrographic seizures were observed thereafter.

Over the course of the six-week admission (Table 1), the respiratory pattern normalized progressively following control of the infectious process and hydrocephalus. At transfer to the general pediatric ward on day 57 of life, neurological examination showed persistent axial hypotonia with mild peripheral hypertonia, without active seizures.

**Table 1:** Clinical timeline of major investigations, interventions, and outcomes during the six-week admission EVD: external ventricular drain

Day	Event
0	Admission at 16 days of life for afebrile seizures and severe hyponatremia; elective preventive intubation; empirical antiseizure and antimicrobial therapy started
1	CRP improved to 55 mg/L
2	Antibiotic therapy narrowed to cefotaxime monotherapy after susceptibility results
3	Extubation; cerebral MRI shows pericerebellar empyema and hydrocephalus with aqueductal obstruction; first EEG shows an irritative background with a burst-suppression pattern
4	Maintenance anticonvulsant therapy switched to levetiracetam
5	CRP rebounds to 424 mg/L; triple antibiotic therapy reinstituted; Biot's breathing identified; first EVD placed
11	Intravenous sodium supplementation discontinued
12	First EVD removed after sterile CSF cultures
14	Second EVD placed for recurrent hydrocephalus
20	CRP normalized
25	Rickham reservoir placed
38	Definitive ventriculoperitoneal shunt with gravitational valve placed
57	Transfer to the general pediatric ward

On the most recent neuropediatric follow-up consultation at 2 years and 4 months of age, the child had a mild-to-moderate global developmental delay (Brunet-Lézine developmental quotient of 77.2, with relative weakness in postural and coordination domains), had acquired independent walking at approximately 19 months, had convergent strabismus of the left eye under ophthalmological follow-up, and had persistent microcephaly. She remained seizure-free since September 2024 on levetiracetam, with a normal 24-hour electroencephalogram in June 2025, and was continuing multidisciplinary rehabilitation (physiotherapy, occupational therapy, and speech therapy) with planned integration into mainstream schooling.

## Discussion

Biot's breathing, also referred to as ataxic respiration, is a rare and often underrecognized respiratory pattern characterized by irregular breathing with unpredictable pauses and variable tidal volumes. First described by Camille Biot [4] in 1876 in a 16-year-old patient with tuberculous meningitis, this breathing pattern was distinguished from Cheyne-Stokes respiration by its irregular rhythm and the absence of a crescendo-decrescendo amplitude pattern. Historically labeled “rythme méningitique,” Biot's breathing was strongly associated with meningitis and medullary dysfunction [5].

Despite its historical significance, Biot's breathing is rarely referenced in contemporary neurological and critical care literature. Several factors contribute to its underrepresentation. First, terminological confusion has emerged over time, with Biot's breathing often conflated with or replaced by more general terms such as ataxic or cluster breathing, resulting in inconsistencies in both recognition and reporting. Second, the advent of widespread mechanical ventilation in critical care settings has limited clinicians' exposure to spontaneous pathological breathing patterns, thereby diminishing familiarity with the sign. Third, in acute and complex clinical contexts, other neurological or systemic signs frequently overshadow subtle respiratory anomalies, leading to underdiagnosis [5].

Physiologically, respiratory rhythm is generated within the dorsal and ventral respiratory groups of the medulla and modulated by pontine and higher cortical inputs; disruption of this circuitry, whether by direct injury or by extrinsic compression such as occuring with obstructive hydrocephalus, produces a spectrum of abnormal breathing patterns, of which Biot's breathing is among the least common but most specific for brainstem or medullary involvement [6,7]. Clinically, the presence of Biot's breathing is therefore considered a sign indicative of brainstem dysfunction, typically resulting from direct injury to the medulla oblongata or secondary to elevated intracranial pressure. In the neonatal setting, particularly in cases of bacterial meningitis complicated by hydrocephalus, the emergence of Biot's respiration may signify brainstem compression or ischemia, as illustrated in our patient by the close temporal association between the appearance of this breathing pattern and the radiological diagnosis of aqueductal obstruction.

Although initially described in the setting of tuberculous meningitis in an adolescent, Biot's breathing has also been reported in the pediatric population in association with acute bacterial meningitis, where its onset likewise coincided with clinical and hemodynamic signs of raised intracranial pressure and prompted urgent osmotherapy [8]. To our knowledge, however, its occurrence has been very rarely documented in neonates, underscoring both the rarity and the diagnostic value of recognizing this pattern in the youngest and most vulnerable patients, whose neurological examination is otherwise limited.

The recognition of Biot's breathing warrants urgent and comprehensive clinical intervention. This includes immediate neurological assessment with neuroimaging to identify structural abnormalities, such as hydrocephalus, and prompt initiation of treatment aimed at reducing intracranial pressure. Supportive respiratory care, including mechanical ventilation when indicated, plays a critical role in preventing secondary hypoxic injury, and management requires a multidisciplinary approach integrating neonatology, neurology, neurosurgery, and infectious disease expertise. In cases complicated by intraventricular or pericerebral empyema, as in our patient, a staged neurosurgical strategy combining external drainage, temporizing reservoirs, and eventual definitive ventriculoperitoneal shunting once CSF parameters normalize has similarly been described as effective in achieving infection control and hydrocephalus resolution [9].

In the present case, the identification of Biot's breathing led to heightened concern for brainstem compromise, prompting timely intervention, including hydrocephalus management and close respiratory monitoring. These measures were followed by the patient's gradual clinical improvement. As this is a single case report, however, a causal relationship between early recognition of Biot's breathing and the favorable outcome cannot be established. The improvement more plausibly reflects the combined effect of infection control and hydrocephalus management, of which recognition of this respiratory sign was one contributing element. At longer-term follow-up, the child showed only a mild-to-moderate global developmental delay despite the severity of the initial presentation, which may reflect the benefit of prompt multidisciplinary management, although continued surveillance remains warranted given the recognized risk of delayed neurodevelopmental sequelae after neonatal bacterial meningitis.

## Conclusions

Biot's breathing is a rare but clinically significant respiratory pattern that should be recognized as a warning sign of brainstem compromise in neonates with bacterial meningitis. In this case, timely identification of Biot's breathing prompted urgent neuroimaging and staged neurosurgical management of hydrocephalus secondary to *Escherichia coli *meningitis complicated by aqueductal obstruction, and was followed by only a mild-to-moderate global developmental delay (Brunet-Lézine developmental quotient of 77.2) at more than two years of follow-up despite the severity of the initial presentation. Clinicians caring for critically ill neonates should maintain awareness of this underrecognized finding, particularly in the setting of infectious central nervous system disease complicated by hydrocephalus.
